# Spectroscopic Studies of the Iron and Manganese Reconstituted Tyrosyl Radical in *Bacillus Cereus* Ribonucleotide Reductase R2 Protein

**DOI:** 10.1371/journal.pone.0033436

**Published:** 2012-03-14

**Authors:** Ane B. Tomter, Giorgio Zoppellaro, Caleb B. Bell, Anne-Laure Barra, Niels H. Andersen, Edward I. Solomon, K. Kristoffer Andersson

**Affiliations:** 1 Department of Molecular Biosciences, University of Oslo, Oslo, Norway; 2 Department of Chemistry, Stanford University, Stanford, California, United States of America; 3 Laboratoire National des Champs Magnétiques Intenses, LNCMI-G, UPR 3228, CNRS, Grenoble, France; University of Wales Bangor, United Kingdom

## Abstract

Ribonucleotide reductase (RNR) catalyzes the rate limiting step in DNA synthesis where ribonucleotides are reduced to the corresponding deoxyribonucleotides. Class Ib RNRs consist of two homodimeric subunits: R1E, which houses the active site; and R2F, which contains a metallo cofactor and a tyrosyl radical that initiates the ribonucleotide reduction reaction. We studied the R2F subunit of *B. cereus* reconstituted with iron or alternatively with manganese ions, then subsequently reacted with molecular oxygen to generate two tyrosyl-radicals. The two similar X-band EPR spectra did not change significantly over 4 to 50 K. From the 285 GHz EPR spectrum of the iron form, a *g*_1_-value of 2.0090 for the tyrosyl radical was extracted. This *g*_1_-value is similar to that observed in class Ia *E. coli* R2 and class Ib R2Fs with iron-oxygen cluster, suggesting the absence of hydrogen bond to the phenoxyl group. This was confirmed by resonance Raman spectroscopy, where the stretching vibration associated to the radical (C-O, ν_7a_ = 1500 cm^−1^) was found to be insensitive to deuterium-oxide exchange. Additionally, the ^18^O-sensitive Fe-O-Fe symmetric stretching (483 cm^−1^) of the metallo-cofactor was also insensitive to deuterium-oxide exchange indicating no hydrogen bonding to the di-iron-oxygen cluster, and thus, different from mouse R2 with a hydrogen bonded cluster. The HF-EPR spectrum of the manganese reconstituted RNR R2F gave a *g*_1_-value of ∼2.0094. The tyrosyl radical microwave power saturation behavior of the iron-oxygen cluster form was as observed in class Ia R2, with diamagnetic di-ferric cluster ground state, while the properties of the manganese reconstituted form indicated a magnetic ground state of the manganese-cluster. The recent activity measurements (Crona *et al.*, (2011) *J Biol Chem* 286: 33053–33060) indicates that both the manganese and iron reconstituted RNR R2F could be functional. The manganese form might be very important, as it has 8 times higher activity.

## Introduction

Ribonucleotide reductases (RNRs) catalyze the reduction of the four ribonucleotides to the corresponding deoxyribonucleotides, providing the precursors for the DNA synthesis and repair in all living organisms [1], [2], [3], [4], [5]. This step is an attractive target for drug design strategies against rapidly proliferating cells such as cancers and various pathogens, as it is the rate limiting step in the DNA synthesis [6]. RNRs are grouped into three classes: I (subclasses a, b, and c), II and III, based on differences in cofactor biosynthesis, oxygen dependency, and quaternary structure [2], [3], [7]. The most prevalent is class I RNR, which is found - with few exceptions - in all eukaryotes, some prokaryotes and viruses [1], [4], [5]. Most class I RNRs are homodimeric complexes (R1 and R2 in class Ia and Ic, and R1E and R2F in class Ib) that assemble into enzymatically active tetramers (R1_2_R2_2_) or higher order oligomers [8], [9]. The R1/R1E subunit contains the active site for reduction of the ribonucleotides, while the R2/R2F subunit contains the di-metal-oxygen cofactor responsible for the formation of the oxygen dependent catalytic tyrosyl radical (Y122^•^ using *Escherichia coli* R2 numbering) [10], [11]. The generated R2 radical is shuttled approximately 35 Å to the active site of the R1 subunit where it forms a thiyl radical, through a proposed conserved network of hydrogen bonded amino acids [4], [7], [12], [13], [14].

**Figure 1:**
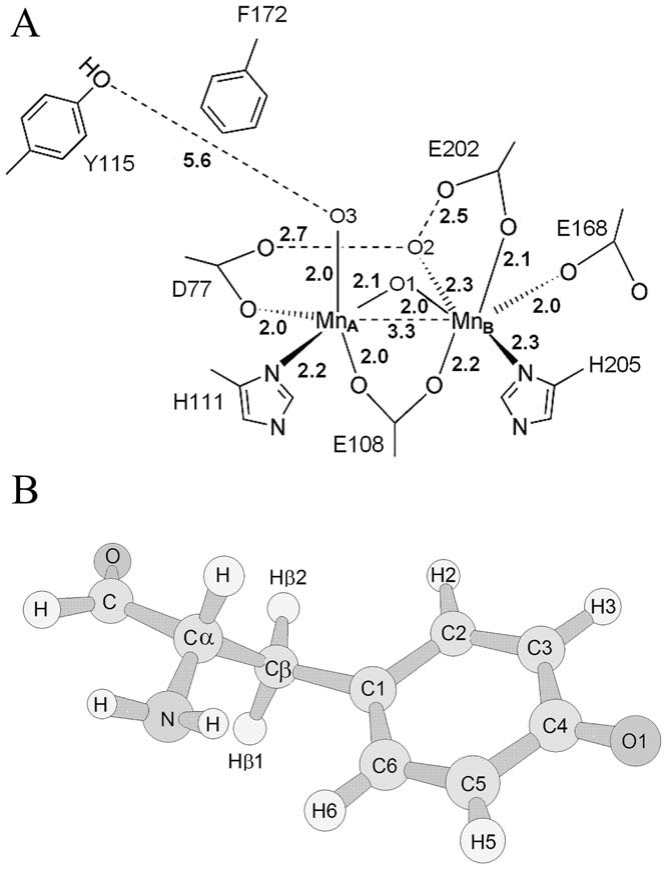
The Mn-substituted R2F-protein from *C. ammoniagenes* (PDB code 3MJO) [**26**] (A). and sketch of themolecular structure of the tyrosyl radical (B). In panel (A) the numbers in bold represent atomic distances (Å). The structure in (B) describes the dihedral angle θ (C6-C1-Cβ-Cα) used to illustrate the orientation of the α-carbon: θ = 0° corresponds to the α-carbon in the plane of the phenoxyl ring, while θ = 90° is perpendicular to the plane.

Division of class I RNR into subclasses Ia-Ic is based primarily on differences in operon structure and metal cofactor [5], [15], [16]. Class Ia RNR is expressed in all mammals, whereas class Ib RNR has only been found in bacteria including pathogenic strains from *Salmonella*, *Bacillus*, and *Mycobacterium* genera. The class Ia R2 protein is only active with a diiron-oxygen cluster, but the class Ib R2F protein can have activity by incorporating manganese or iron clusters [17], [18], [19]. Recently, several studies support manganese as the physiologically relevant class Ib cofactor for RNR metabolism [20], [21] and *B. subtilis* class Ib RNR is possibly a dimanganese(III)-Y• enzyme [22]. From activity studies, the manganese reconstituted protein showed a higher specific activity relative to iron [20]. The Fe^III^_2_-Y^•^ cofactor can be generated by self-assembly with Fe^II^ and O_2_, but formation of the Mn^III^_2_-Y^•^ cofactor requires the additional presence of a flavodoxin like protein, NrdI [20], [23], [24], [25]. The Mn^III^_2_-Y^•^ cofactor structure has recently been solved for class Ib R2F from *Corynebacterium ammoniagenes*, see Figure 1A [26]. The newest subclass is Ic; identified in *Chlamydia tractomatis* and several Archaea. These utilize a Mn-Fe cofactor and lack the catalytic tyrosine residue [15], [27], [28], [29].

The *Bacillus* genus contains at least six different pathogens with a close genetic relationship among the group, however a high diversity in virulence is present [30]. *B. cereus* is an opportunistic pathogen that is commonly isolated from food and causes food poisoning. Differences in the pathogen and host RNRs could be exploited for drug design and the development of alternative antimicrobial agents. Thus, detailed molecular level descriptions are of high interest. For instance, reduction of the tyrosyl radical to tyrosine is part of the action mechanism of several current RNR-targeting drugs and understanding differences between host and pathogen radicals could lead to better pharmaceuticals [6], [31], [32]. Electron Paramagnetic Resonance (EPR) studies of the tyrosyl radical in various RNR class Ib R2F proteins describe differences in the orientation of the tyrosine ring plane (see Figure 1B for definition of different angels), relative to the class Ia RNR R2, which result in a large spread of the hyperfine tensor values of the tyrosyl-radical protons [1], [12], [33], [34], [35]. There is relatively low sequence identity between the RNR proteins from the *B. cereus* group and the other class Ib RNR from *E. coli* and *C. ammoniagenes* (∼40%) [35], [36]. In this work, we use electronic and magnetic spectroscopic signatures of both iron and manganese reconstituted tyrosyl radical of *B. cereus* to obtain a molecular level description of the electronic properties associated with the radical/metals sites. This study provides nice examples of the differences in the electronic/magnetic properties of the catalytic/oxygen binding site in RNR proteins, even within the same subclass.

## Materials and Methods

Commercial agents were used for chemicals (as obtained): 4(-2hydroxyethyl)-2-piperazineethanesulfonic acid (HEPES) (Sigma), tris(hydroxymethyl)-aminomethane (Tris) (Sigma), ferrous ammonium sulphate hexahydrate (Merck), ampicillin (Sigma), manganese(II) chloride tetrahydrate (Merck), DNeasy (Qiagen), pET-22b plasmid (Novagen), BL21 (DE3) Gold cells (Stratagene), deuterium oxide (99.9%D, Cambridge Isotope Laboratories), H_2_^18^O(l) (Cambridge Isotope Laboratories, 97%), ^18^O_2_ (g) (Cambridge Isotope Laboratories, 97%), and hydroxyurea (HU) (Sigma). Deuterated buffer was degassed with 99.9% pure argon for at least 1 h.

### Protein expression and purification

*B. cereus* R2F, NrdI, and the thioredoxin like protein, BC3987 (and its thioredoxin reductase), were expressed and purified as previously described [24], [37], [38]. The extinction coefficient was determined using the Edelhoch method [39].

### Sample preparation

After purification, the RNR R2F protein was pretreated with 10 mM hydroxyurea and 5 mM EDTA at 4°C for 20 min and passed over a 5 mL HiTrap desalting column (GE healthcare) to remove the iron and to reduce the tyrosyl radical before reconstitution with Fe^II^. The iron-tyrosine radical site in *B. cereus* R2F was generated by addition of excess Fe^II^ and O_2_ and incubated at 0°C for 10 minutes. The final volume of the EPR samples was 180–200 µL in 50 mM HEPES, 100 mM KCl, and 20% glycerol (v/v), pH 7.5. Samples of active *B. cereus* R2F for resonance Raman were prepared identically, but protein was diluted into 50 mM Tris-HCl, 100 mM KCl, and pH 7.5. Deuterated resonance Raman samples were prepared by diluting and re-concentrating apo protein in pure D_2_O (99.9%D, Cambridge Isotope Laboratories) and then in 50 mM Tris-HCl, 100 mM KCl, pD = 7.9 using Amicon Ultra-15 (Millipore) 7 times. ^18^O samples were prepared by incubating for approximately 45 min in H_2_^18^O after formation of the radical [40]. The *in vitro* generation of the dimanganese-Y^•^ cofactor was carried out as previously described using NrdI_hq_ (hydroquinone form of NrdI) in the reacting the mixture with oxygen [20]. After tyrosyl-radical formation the HF-EPR sample, was additionally passed over a 5 mL HiTrap desalting column (GE healthcare) to remove most of the Mn(II) not in di-Mn cluster and finally the sample was concentrated to about 200 µl by centrifugation on a Millipore/Amicon microcon YM-30.

### UV/Vis spectrophotometric assays

Light absorption spectra were measured on a Hewlett-Packard 8452 diode array spectrophotometer in the wavelength range of 250–700 nm using 1 cm optical-path quartz cuvette.

### X-band EPR experiments

EPR spectra were recorded at X-band on a Bruker Elexsys 560 EPR spectrometer fitted with a Bruker ER41116DM dual-mode cavity and using a He-flow cryostat (ESR 900, Oxford Instruments). The sample spin concentration was obtained by comparing double integrals of spectra with 1 mM Cu^II^ EDTA prepared in a solution of 50 mM HEPES, pH 7.5, 20% (v/v) glycerol, recorded under non-saturating conditions. All spectra were measured under identical non-saturating microwave power. First-derivative EPR spectra were recorded at different microwave power (*P*) and at various temperatures to determine the microwave power at half saturation (*P_1/2_*) for each temperature examined. The data were fitted with the Equation 1 [41], [42], [43].
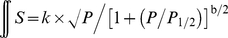
(1)The term ∫∫*S* represents the double integrated signal intensity (S), *P* is the applied microwave power, *b* is the relaxation factor (*b* = 1 for inhomogeneous line broadening and *b* = 3 for homogeneous line broadening) [41], [42], *P*_1/2_ is the power at which the signal is half-saturated and *k* is an experimental constant associated with the instrument. In order to apply Eq. 1 the following experimental conditions must be satisfied: i) the samples should be in a region of the cavity with maximum microwave field (*H*_1_), thus the filling factor needs optimization, ii) the sample temperature must be constant and iii) the frequency and the gain must also be constant. Eq. 1, however, is not strictly applicable when dipolar couplings and/or exchange interactions are effective in the system. This is due to the fact that the half-saturation power (*P*_½_) is defined by Eq. 2 and Eq. 3 where *V* represents the cavity volume, *Q* the cavity quality factor (*Q* = *H*_1_^2^ *V*/2*P*), *P* the power dissipated in the cavity and *γ* the gyromagnetic ratio. Eq. 2 assumes that all spins at resonance saturate equivalently, hence they feature the same product (*T*_1_ *T*_2_).
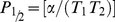
(2)

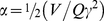
(3)When dipolar and/or exchange interactions are present in the spin system, the product (*T*_1_ *T*_2_) is not constant thus 1≤*b*≤3 is no longer valid and *b* becomes less than 1 [44], [45], [46]. The simulated EPR spectra were computed with two programs. (i) The Bruker Biospin XSophe V.1.1.4 (matrix diagonalization, *g*-strain, where the strain is defined as Δ*g* = *g*e µB/h×(ΔB/ν), with strain imposed Δ*g*_(x,y,z)_ = 0.0001). (ii) The simulation platform SimFonia (Bruker biospin, V. 1.25), which uses powder perturbation theory but without additional contributions to signal broadening due to *g*-strain effects. We observed that with X-Sophe (*g*-strain model), we could simulate by using the same line-width tensor both X-band and High-Field EPR spectra. The use of the same line-width tensor as employed in the X-Sophe platform by SimFonia did not allow to reproduce satisfactorily the observed X-band spectrum of Fe *B. cereus* RNR R2, and only a much larger line-width tensor (L_x,y,z_ as shown in Table 1) gave comparable well reproduced spectrum (see Figure S1). We did not use the software SimFonia for simulation of the High-Field EPR spectrum of Fe loaded RNR R2F form. Estimation of the θ angle, namely the angle between tyrosyl plane versus plane generated by the ethyl residue (Figure 1B) can be obtained following two approaches; (1) Through simulation under spin-Hamiltonian approximation the best set of anisotropic proton hyperfine couplings (H-*hfc*) (*A*) terms for the H_β1_ and H_β2_ protons are obtained, which can be used to fed the *hfc* H_β1_, H_β2_ (Y) *vs* θ (X) diagram derived by theory by Himo and Gräslund [47], or (2) by using the empirical relation developed by Svistunenko and Cooper [48] where only two input variables θ and *ρ*_C1_ (McConnell spin density) can be used to simulate the entire EPR envelope; for example, for a fixed combination of θ- and *ρ*_C1_-values, *A*_(β1)_iso and *A*_(β2)_iso can be extracted and compared with those determined experimentally. In this work, for estimation of θ we did use the first approach (1). Sign and magnitude of *hfc* terms for H_β1_, H_β2_, H3 and H5 were initially derived from DFT analyses H_β1_, H_β2_ *hfc* values were then compared to those computed by Himo and Gräslund [47], followed by stepwise deconstruction of the EPR resonance line, so to refine the anisotropic H-*hfc* components for H3 and H5 protons that generated a simulated EPR envelope consistent with the recorded experimental spectra.

**Table 1:** The *g–tensors*, *hfc–tensors* (A, mT), linewidths (LW, mT), and rotational θ angle (°) for the tyrosyl radical in class Ia and class Ib RNRs and the Y_D_ radical in photosystem II, *Inequivalent C_3_ and C_5_ protons, **Using XSophe (v. 1.1.4) (matrix diagonalization, g-strain model).

EPR parameters		RNR class Ia		RNR class Ib				PS II Y_D_
		*E. coli*	Mouse	*B. cereus*	*B. anthracis*	*S. typhimurium*	*M. tuberculosis*	
**g_x_**		2.0090	2.0076	2.0090 (2.0094)	2.0100***	2.0089	2.0092	2.0076
**g_y_**		2.0044	2.0043	2.0044 (2.0039)	2.0042***	2.0043	2.0046	2.0043
**g_z_**		2.0021	2.0022	2.0021 (2.0022)	2.0026***	2.0021	2.0022	2.0022
**H_3,5_**	**A_x_**	−0.96	−0.91/−0.73*	−0.91 (−0.78)	−0.91	−1.15	−1.17	−0.91
	**A_y_**	−0.28	−0.44/−0.48*	−0.49 (−0.42)	−0.48	−0.25	−0.19	−0.26
	**A_z_**	−0.70	−0.66/−0.58*	−0.79 (−0.69)	−0.79	−0.71	−0.71	−0.70
**Hβ_1_**	**A_x_**	1.96	2.14	1.68 (1.56)	1.68	1.05	0.89	0.72
	**A_y_**	2.12	1.90	1.49 (1.35)	1.48	0.74	0.73	1.05
	**A_z_**	1.96	2.15	1.55 (1.45)	1.55	0.95	0.90	0.72
**Hβ_2_**	**A_x_**	0.18	0.95	0.20 (0.23)	0.20	<0.3	-	0.19
	**A_y_**	−0.07	0.25	0.02 (0.06)	0.02	<0.3	-	0.19
	**A_z_**	−0.07	0.57	0.02 (0.08)	0.02	<0.3	-	0.51
**LW_x_**		-	0.45	0.34 (0.29)	0.64	0.58	0.58	-
**LW_y_**		-	0.35	0.31 (0.39)	0.63	0.53	0.54	-
**LW_z_**		-	0.44	0.30 (0.29)	0.48	0.37	0.29	-
**Θ**		30	35	60 (65)	60	75	75	82
**Ref.**		[54], [58], [77]	[51]	This work**	[35]	[33]	[56]	[33]

Note: similar results can be obtained with SimFonia software (v. 1.25) using the same *g–tensors*, *hfc–tensors* but very different line-width tensor (for the Fe reconstituted form LWx,y,z = 0.560, 0.630, 0.490, Lorentzian/Gaussian line-width = 0.63; for the Mn reconstituted form LWx,y,z = 0.570, 0.795, 0.550. Lorentzian/Gaussian line-width = 1). In *B. Cereus*, the sign and magnitude of the anisotropic hyperfine tensor components are taken from DFT analyses. The definition of θ is given in Figure 1B.

*** The g-tenors for *B. anthracis* R2F are estimated values from simulations. The values in parenthesis ( ) corresponds to the tyrosyl radical in *B. cereus* R2F reconstituted with Mn.

### High-field EPR measurements

The low temperature 285 GHz (high-field/high-frequency EPR, HF-EPR) spectra were obtained with a 95 GHz Gunn oscillator (Radiometer Physics, Germany) coupled to a frequency tripler as the frequency source and a superconducting magnet with a maximum field of 12 T at 4.2 K (Cryogenics Consultant, UK) for the main magnetic field. Temperature of sample was changed by a variable temperature Insert (Oxford Instruments, UK) over a temperature range from 1.5 K to 300 K, with the sample directly in the helium flux. The detection of the light transmitted through the sample was performed with a ‘hot electron’ InSb bolometer as described [33], [49].

### Computational procedures

The theoretical modeling of the tyrosyl radical was performed in the gas phase by density functional theory (DFT) considering the radical in the uncharged form (neutral) with the spin unrestricted B3LYP functional (Exchange of 0.2000 Hartree-Fock, 0.0800 Slater and 0.7200 Becke, correlation of 0.8100 LYP and 0.1900 VWN1RPA) using the Euler-Maclaurin-Lebedev (EML grid; 70,302) quadrature formula and basis set 6-311++G(d,p) as implemented in the computational package Spartan 10. The molecular structure of the tyrosyl molecule was optimized under constrained θ (C6-C1-Cα-Cβ) torsional angle (root mean square gradient below 10^−7^), followed by frequency calculation to derive vibrational frequencies. Single point calculations were carried out on the optimized geometry with Gaussian 03 (Version 6.0) using the same functional but tighter SCF convergence (SCF = tight) to obtain accurate Mulliken atomic spin-population analyses. The list of vibrational frequencies (cm^−1^) and zero point vibrational energies are provided in the Calculation S1.

### Resonance Raman spectroscopy

Resonance Raman spectra were recorded using a Spex 1877 CP triple monochromator with 1200, 1800, and 2400 grooves/mm holographic gratings and an Andor Newton CCD detector cooled at −80°C. Excitation was provided by either a Coherent I90C-K Kr+ ion laser (λ_exc_ = 406.7 nm) or an Innova Sabre 25/7 Ar+ CW ion laser (λ_exc_ = 379.5 nm). The spectral resolution was ∼2 cm^−1^. Spectra were recorded on sampled cooled in a finger Dewar with liquid nitrogen (77 K) at a power of 5 mW (379.5 nm) or 10 mW (406.7 nm) at the sample. Reported spectra are the sum of 30 to 60 accumulations of 10 to 30 seconds and 5 independent measurements using full vertical binning on the CCD. Baseline spectra were collected using ground activated charcoal, buffer and apo protein. The rRaman spectra were also collected with a Jobyn Yvon Horiba T64000 instrument equipped with a 410 nm Kaiser Optical holographic Super-Notch filter to serve as a single spectrograph to avoid high losses of Raman light. Here a three stage laser system was used as the light source: A Spectra-Physics Millennia Pro 12sJS Nd∶YAG solid state laser (6.5 W at 532 nm) pumped a Sirah Matisse TR Ti∶Sa ring Laser that produced 1 W at 820 nm. This was finally doubled to 410 nm in a Spectra-Physics Wavetrain frequency doubler (550–990 nm) to yield 20 mW of laser light with a line width of better than 4 MHz. The power of the 410 nm laser at the sample was ∼5 mW due to losses at instrument mirrors and lenses. The low band width and the small shifts of the Raman peaks investigated required an entrance slit width of 100 µm and a grating with 3600 grooves per mm to ensure sufficient resolution. 60 scans of 60 seconds were averaged for each Raman spectrum. The fluorescence signal that inevitably occurred was subtracted by fitting with a polynomial function, and the frequency scale was calibrated using 4-acetamidophenol. This resulted in a final precision that was ∼2.5 cm^−1^ over this region. The reference peak values were obtained from literature tables [50].

## Results and Discussion

### Reconstitution of the tyrosyl radical in *B. cereus* R2F

**Figure 2:**
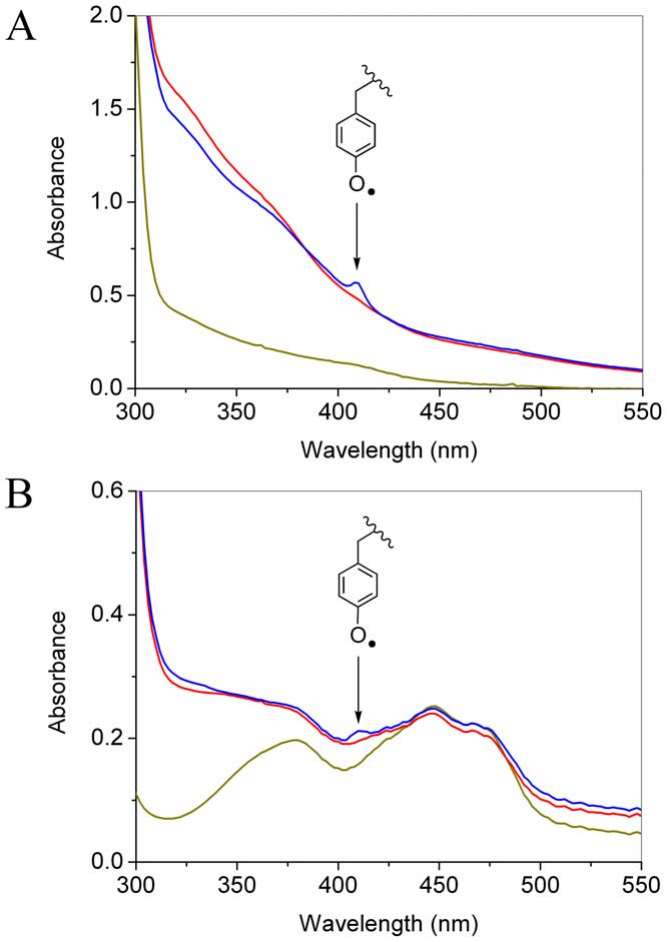
UV-Vis spectra of reconstituted R2F. (A) UV-Vis spectrum of 75 µM R2F prior to reconstitution (dark-yellow line), R2F reconstituted with ferrous iron (blue line), and after incubation with 4 mM HU for 5 min (red line) at room temperature. (B) UV-Vis spectrum of 50 µM Mn^II^_2_-R2F reconstituted with 25 µM of NrdI_hq_ and O_2_ (g) (blue line), 50 µM Mn^II^_2_-R2F with 25 µM of NrdI_ox_ (dark-yellow line), and manganese reconstituted R2F treated with 4 mM HU for 5 min at room temperature (red line).

The “apo” *B. cereus* R2F (Figure 2A, dark yellow line) can be readily reconstituted with excess ferrous iron and O_2_ (Figure 2A, blue line). Inductively coupled plasma (ICP)-analysis demonstrated that “apo” protein contains ∼0.25 equivalents of iron per dimer subsequent to treatment with chelating agents [37]. Circular dichroism (CD) studies prove that this spurious iron bound to the protein does not form the dinuclear metal cluster and does not take part in the process of tyrosyl radical formation after oxygen binding and oxidation of the active dinuclear metal core [37]. The UV-visible spectrum of the iron loaded R2F protein (radical and oxy form, active) displays a sharp peak at 408 nm and broader bands at ∼325 and 365 nm. The former signal is characteristic of a tyrosyl radical and the latter signals of a di-iron-oxo cluster (Figure 2A, blue line). The maximum yield of tyrosyl radical was obtained with 7 equivalents of ferrous iron per R2F dimer. From EPR, the radical yield is ∼1 per dimer (see below). From UV-vis absorption the radical concentration was estimated as ∼0.8 per dimer. The discrepancy in the estimated radical content could arise from the difference in protein concentrations used in the two methods (50–75 µM for UV-vis and 200 µM for EPR). The tyrosyl radical in the active (oxy) Fe loaded protein was relatively stable and its absorption feature did not change during 30 min of incubation at room temperature. The radical signal completely vanished after 5 min when the active (oxy) Fe loaded protein was treated with 4 mM hydroxyurea (HU), which is a common radical scavenger for RNR R2 (Figure 2A, red line).

The tyrosyl radical in *B. cereus* R2F can also be formed when manganese metal ions occupy the oxygen binding site, but only in the presence of NrdI, as recently shown for *E. coli* class Ib R2F [20], [21] and *C. ammoniagenes* RNR [26]. Mn^II^_2_-R2F was initially incubated anaerobically with the hydroquinone form of NrdI (NrdI_hq_). When the sample was exposed to O_2_ gas, a sharp tyrosyl radical-type absorption feature at 408 nm appeared (Figure 2B, blue line) in addition to the generation of oxidized NrdI (NrdI_ox_) (Figure 2B, dark yellow line). Maximum radical formation was obtained after incubation with 4 equivalents of Mn(II) per R2F dimer, monitored at 408 nm. The radical signal decayed during incubation, both at room temperature and on ice, and about half of the radical UV-vis absorption is lost (result not shown) after 30 minutes on ice temperature (T∼4°C). When the oxidized Mn(III) cluster R2F-Tyr^•^ was incubated with HU for 5 min, the radical absorption signature at 408 nm disappeared (Figure 1B, red line). Control experiments show that the 408 nm does not originate from NrdI_ox_, which is included in the reconstitution (Figure 2, dark-yellow line). These results clearly indicate that R2F tyrosyl-radical can be generated successfully with NrdI_hq_. Finally, the manganese loaded R2F-Tyr^•^ protein is less stable then the iron loaded R2F-Tyr^•^ form, both at room and at ice temperatures.

### Low temperature electron paramagnetic resonance studies of *B. cereus* R2F tyrosyl-radicals

**Figure 3:**
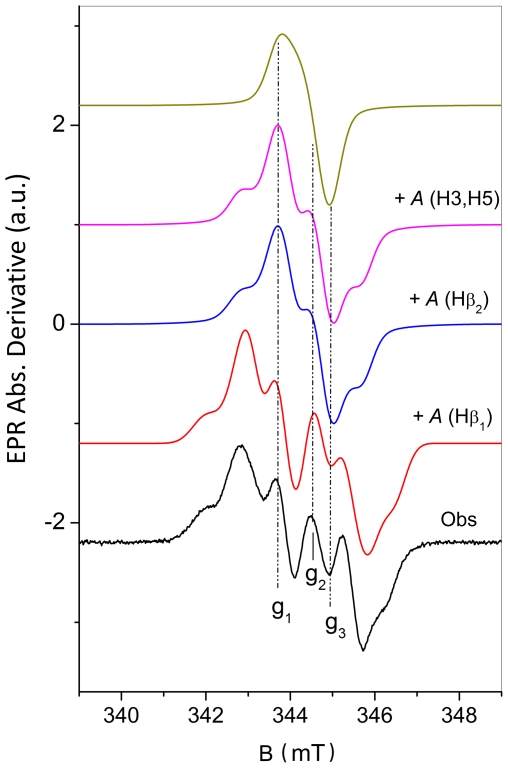
X-band (9.66 GHz) EPR spectrum of the R2F-Fe^III^_2_-Tyr^•^ from *B. cereus* R2F (200 µM) recorded at T = 20 K, 12 µW microwave power, 100 KHz modulation frequency, 0.2 mT modulation amplitude and 2 scans (Obs). The spectrum simulation (traces olive, purple, blue and red) show the EPR resonance line where only the anistropic *g*-tensor components are considered (olive line), when contribution from anisotropic hyperfine terms (*A*) due to the two H3, H5 protons (purple line, +*A-*H3, H5) are included, by adding further contribution arising from the Hβ_2_ proton (blue line, +*A-*H3, H5+*A*-Hβ_2_), and the final simulation containing all relevant contributions to the EPR envelope (red line, +*A*-H3, H5+*A*-Hβ_2_+*A*-Hβ_1_). List of simulation parameters (*g*-tensor, *A*-tensor, Line-width tensor) are given in Table 1).

**Figure 4:**
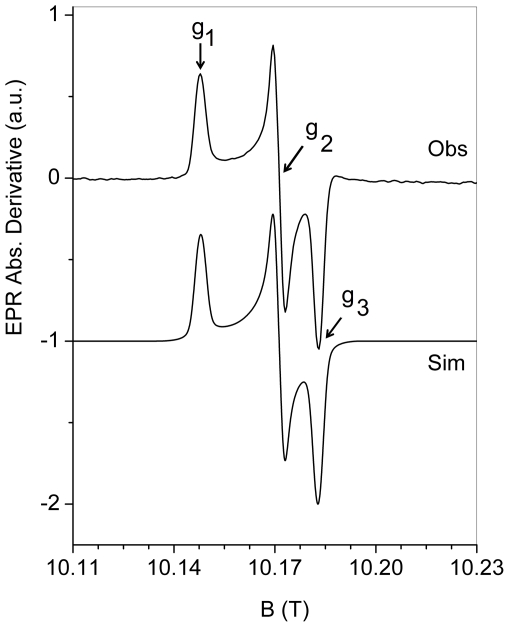
HF-EPR (285 GHz) spectrum of R2F-Fe^III^_2_-Tyr^•^ from *B. cereus* R2F (200 µM) recorded at T = 5 K, with a modulation amplitude of 1.5 mT (Obs) and its spectrum simulation (Sim).

**Figure 5:**
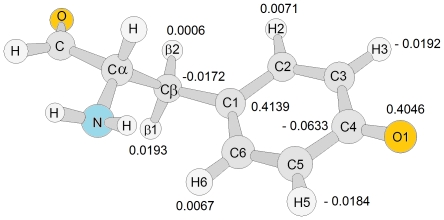
Spin-density distribution of an isolated tyrosyl radical obtained by density functional theory (DFT/UB3LYP/6-311++G(d,p) in gas phase, neutral form after geometry optimization (<S^2^> = 0.7507, the dihedral angle θ was constrained to 60°). In this constrained conformation, the calculated atomic spin densities (from Mulliken population analyses) reveal much larger positive values located on H(β1) with respect to the H(β2) proton.

Formation of a tyrosyl radical occurs together with metal-cluster oxidation. The electronic/magnetic fingerprints of the formed tyrosyl radicals were assessed with low-temperature EPR spectroscopy. Differences in the radical resonance envelope with different Fe^III^_2_-R2F-Tyr^•^ or Mn^III^_2_-R2F-Tyr^•^ cofactors were observed. The tyrosyl radical in R2F-Fe^III^_2_-Tyr^•^ was measured over a temperature range 4–77 K with two EPR microwave frequencies 9.6 (X-band) and 285 GHz (HF-EPR). The spectra are shown in Figure 3 (X-band, T = 20 K, lower black line, Obs) and Figure 4 (HF-EPR, T = 5 K, upper line, Obs) together with simulations. The anisotropic *g*-tensor components associated with the tyrosyl radical are poorly resolved at X-band frequency (Figure 3), however, they are clearer at high-frequency (Figure 4, T = 5 K) with *g*_1_ = 2.0090, *g*_2_ = 2.0044, and *g*_3_ = 2.0021. The X-band EPR spectrum from R2F-Fe^III^_2_-Tyr^•^ of *B. cereus* exhibits similar resonance signature to that observed for the close homologue *B. anthracis* R2F [35]. Both have an EPR spectral width of ∼6.0 mT. Similar to *B. anthracis* R2F, the EPR signal in *B. cereus* R2F displays several well resolved anisotropic hyperfine splitting components (*hfc*) from the magnetic interaction of the unpaired tyrosyl radical spin with magnetically non-equivalent hydrogen nuclei of the tyrosine backbone. The 285 GHz spectrum does not show resolved *hfc* even using 0.4 mT modulation amplitude. Poor resolution of the *hfc* at high-fields has been observed previously in the HF-EPR spectrum of mouse R2, which contains even stronger H-*hfc* terms [51], [52]. It has been well established, both theoretically and experimentally, that the rotational configuration of the tyrosyl ring, namely the dihedral angle (θ) (Figures 1B and 5, see earlier description in materials and methods), strongly influences the values of the H-*hfc* in the tyrosyl backbone, which also depends on spin-density distribution of the tyrosyl radical specially on C1 [53]. Thus, in view of the similarities observed in the EPR envelope with *B. anthracis* R2F, the dihedral angle (θ) of the tyrosyl radical in *B. cereus* R2F was estimated as ∼60° [47], [48]. This conformation accounts well for nearly identical spin-density (from Mulliken population analysis) located on the H3,H5 protons, and for the large difference in spin-density between the β_1,2_-protons (Figure 5). The derived EPR parameters are collected in Table 1 together with *g*, *A* and θ values for the tyrosyl radicals in RNR R2 from *E. coli* R2 [52], [54], mouse R2 [34], [49], [51], [52], [55], *B. anthracis* R2F [35], *S. typhimurium* R2F [33], *M. tuberculosis* R2F [56] and photosystem II (PS II) Y_D_ [33], [57]. The estimated angle (θ) in *B. cereus* Fe^III^_2_-R2F differs significant from those observed in the other class Ib (θ = 75°) and class Ia (θ = 30–35°) RNRs (see Table 1) [33], [35], [47], [48], [55], [56]. However, the *g*_1_-value observed in *B. cereus* R2F is similar to those found in class Ib R2F proteins and class I *E. coli* R2 [33], [54], [56], [58]. Observation of high *g*_1_-value suggests that the radical moiety is not involved in a hydrogen bonding interaction with nearby groups such as water molecules. For further discussion of hydrogen bond effects, on tyrosyl-radicals *g*_1_-value and changes in spin densities between C4 and O1(Figure 1B), we refer to the following references [48], [52], [54], [55], [59], [60]. The spin concentration (*S_c_*) of the tyrosyl radical was ∼1 spin per dimer. Moreover, *S_c_* remained nearly constant throughout the temperature range examined (T = 4–77 K).

**Figure 6:**
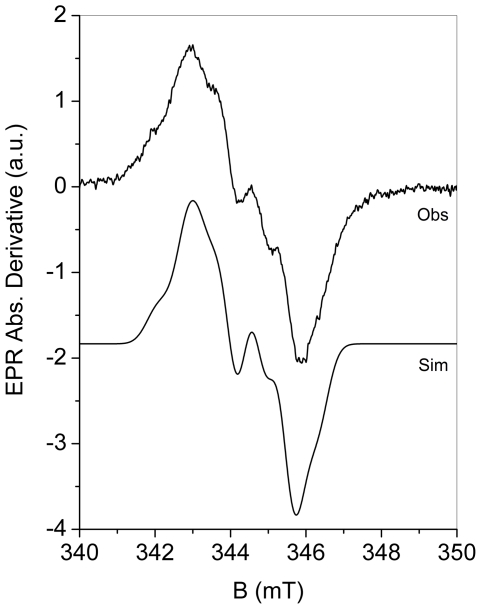
The X-band (9.66 GHz) EPR spectrum of R2F-Mn^III^_2_-Tyr^•^ *B. cereus* R2F (200 µM R2F) reconstituted in presence of 2X NrdI_hq_. Recorded at T = 20 K, 16 µW microwave power, 0.2 mT modulation amplitude, and 4 scans (Obs) and its spectrum simulation (Sim).

The X-band EPR spectrum of the tyrosyl radical in R2F-Mn^III^_2_-Tyr^•^ *B. cereus* R2F recorded at (T = 20 K) is shown in Figure 6 (upper line, Obs) together with one simulation (lower line, Sim). The recorded resonance and derived g-tensor parameters (using spectrum simulation, *g*_1_ = 2.0094, *g*_2_ = 2.0039, *g*_3_ = 2.0022), thus are only tentative but show alteration of the magnetic fingerprints of the tyrosyl radical when manganese-ions replaces two iron ions. Furthermore, the loss of resolved A_H_-hyperfine resonance signals accompanied by signal broadening (∼8.5 mT) is evident in the R2F-Mn^III^_2_-Tyr^•^ spectrum. The list of R2F-Mn^III^_2_-Tyr^•^ EPR simulation parameters (values enclosed in parentheses, tentative) are given in Table 1. The dihedral angle (θ) of the tyrosyl radical in *B. cereus* R2F-Mn^III^_2_-Tyr^•^ was estimated as ∼65°. The spin concentration of the tyrosyl radical was determined in this case as ∼0.25 spin per dimer. The *g*_1_ resonance feature of the *B. cereus* R2F-Fe^III^_2_-Tyr^•^ HF-EPR signal can also be observed in the HF-EPR spectrum of R2F-Mn^III^_2_-Tyr^•^ (Figure S2, T = 5 K). However, the entire *g*-tensor values of R2F-Mn^III^_2_-Tyr^•^, estimated from X-band measurements, is poorly resolved in the HF-EPR spectrum. This is due to low radical concentration and the presence of manganese impurities overlapping on *g*_2_ and *g*_3_. HF-EPR is very sensitive to Mn(II) impurities and removal of most of the Mn(II) by gelfiltation was needed, which lower the tyrosyl-radical content in R2F-Mn^III^_2_-Tyr^•^ and at 5 K the two Mn(II) impurities (Figure S2) are partially saturated. At 10 K or 15 K the R2F-Mn^III^_2_-Tyr^•^ radical 285 GHz spectrum, is much more dominated by the Mn(II) impurities. Our findings indicate that presence of Mn(II) impurities can only be partly removed from the sample, but at the cost of a substantial loss in radical yield and resolution of the g-tensor.

In the Mn reconstitution process, the use of NrdI_ox_ was found to be ineffective, and only the use of NrdI_hq_ lead to successful loading of Mn into the protein active site competent for oxygen activation and formation of tyrosyl radical. The X-band double integrated EPR signal of R2F-Mn^III^_2_-Tyr^•^ followed the Curie-law and furthermore the spectra recorded at T = 50 K exhibited similar resonance envelope as those observed at lower temperatures without signal- broadening. The tyrosyl radical signal of R2F-Mn^III^_2_-Tyr^•^ shown in Figure 6 was obtained after EDTA treatment under anaerobic conditions of apo-R2F and followed by oxygen exposure before sample measurement. From a chemical perspective, the tyrosyl radical in R2F-Mn^III^_2_-Tyr^•^ appeared less stable than in R2F-Fe^III^_2_-Tyr^•^, and the radical fingerprints (UV/Vis and EPR) degraded rapidly when the protein samples were not quickly frozen in liquid N_2_. The overall radical X-band EPR resonance envelope has Curie-type temperature behavior and no modification of the EPR signal width from 4 K to 50 K in *B. cereus* R2F-Mn^III^_2_-Tyr^•^, render the magnetic properties of the R2F-Mn^III^_2_-Tyr^•^ system in *B. cereus* different from those reported for other manganese forms, in *E. coli* and in *C. ammoniagenes* RNR R2F [20], [26]. In these later R2F proteins, the X-band EPR resonances changed significantly with the temperature, and contained a so-called “split-signal” arising from a strong magnetic interaction with the dinuclear Mn^III^ cluster that collapsed at T>30 K [20], [26]. Importantly, the total spectrum-width (X-band) in *C. ammoniagenes* RNR R2F was as large as 40 mT at T = 20 K, while in *B. cereus* the resonance envelope is less wide, at least by a four fold factor. Thus, the magnetic interaction between the tyrosyl radical and dimanganese catalytic core in *B. cereus* R2F must not be as strong as in *E. coli* and *C. ammoniagenes* R2Fs. The observed limited spectral broadening is consistent with the presence of a small, through-space, dipolar contribution. Even though the X-band EPR spectral simulation does not reproduce exactly the observed spectrum-width (Figure 6), the main resonance features are convincingly reproduced. The derived A_H_ terms for the tyrosine protons backbone and the overall EPR envelope suggest electronic and/or conformational changes in the tyrosyl radical in R2F-Mn^III^_2_-Tyr^•^ and slightly ∼5° θ (Table 1) larger than in R2F-Fe^III^_2_-Tyr^•^. As described in the following section, such differences between the tyrosyl radical with nearby manganese or iron metal ions, appear also in the microwave power saturation behaviors of R2F-Mn^III^_2_-Tyr^•^ and R2F-Fe^III^_2_-Tyr^•^, as observed by both the P_1/2_ and *b* values.

### Differences in X-band EPR microwave power saturation properties of tyrosyl radical in *B. cereus* R2F reconstituted with Fe or Mn

**Figure 7:**
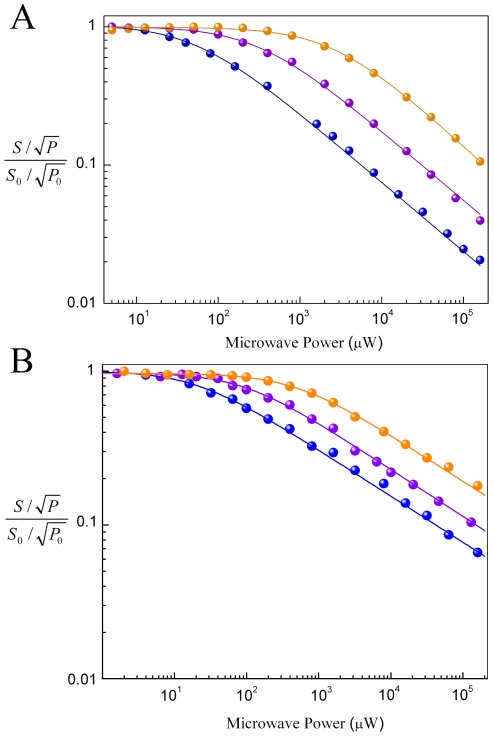
The Microwave power saturation of the iron reconstituted tyrosyl radical in *B. cereus* R2F recorded at T = 20 K (blue circles), T = 50 K (violet circles) and at T = 100 K (orange circles) (A). The factor ∫∫*S* represents the double integrated EPR signal intensity, *P* the applied microwave power and ∫∫*S*_0_ the double integrated EPR signal intensity recorded at the lowest microwave power *P*_0_. The correspondent simulations of the experimental data are shown by solid lines. The *b*-values were equal to 1 for T = 20 K and 50 K and *b* = 0.9 for T = 100 K. P_1/2_ (20 K) = 0.05 mW, P_1/2_ (50 K) = 0.3 mW, P_1/2_ (100 K)≥2.6 mW. Panel (B) shows the microwave power saturation of the manganese reconstituted tyrosyl radical in *B. cereus* R2F recorded at T = 4 K (blue circles), T = 20 K (violet circles), T = 50 K (orange circles). The *b*-values were found equal to 0.6 for all the temperatures examined. P_1/2_ (4 K) = 0.02 mW, P_1/2_ (20 K) = 0.10 mW, P_1/2_ (50 K) = 0.46 mW.

Analysis of progressive microwave power saturation of the EPR spectra at different temperatures, even without employment of relaxation enhancement in the saturation-recovery experiment, can provide insights into the relaxation properties of the radical system and its surrounding, as discussed in the seminal works of Portis [42], Castner [41] and Sahlin *et al.* [61], and well summarized recently by Hirsh and Brudvig [45]. The microwave power saturation results of R2F-Mn^III^_2_-Tyr^•^ and R2F-Fe^III^_2_-Tyr^•^ proteins were compared with those previously derived from different class Ia and Ib R2/R2F proteins. The microwave power saturation experiments are sensitive to interactions between a radical unit and a fast-relaxing metal site. The presence of effective magnetic interaction should change the radical relaxation properties, due to changes in T_1_ (spin-lattice), T_2_ (spin-spin) or both. *P_1/2_* and *b* become informative parameters in this case, *b*-values tend to converge to values smaller than 1 as a result of dipolar and exchange contributions (hence relaxation is neither purely homogeneous or purely inhomogeneous), while the half-saturation value (*P_1/2_*), directly linked to *1*/(*T*_1_ *T*_2_), is expected to become larger than that observed in absence of magnetic interaction. The microwave power saturation trends observed at different temperatures for R2F-Fe^III^_2_-Tyr^•^ are shown in Figure 7A. The calculated *b* value is 1 (inhomogeneously broadened line) and the *P_1/2_* -value at T = 20 K (P_1/2_ = 0.05 mW) was found lower than that obtained from *B. anthracis* R2F, *M. tuberculosis* R2F, and mouse R2, which all exhibit a *P_1/2_*-value of ∼0.1 mW at T = 20 K [35], [56], [61]. However, the *P_1/2_*- value of R2F-Fe^III^_2_-Tyr^•^ in *B. cereus* was slightly larger than in *E. coli* R2 (*P_1/2_* = 0.03 mW) at the same temperature [35], [61]. At higher temperatures (T = 50 K and T = 100 K), the *P_1/2_*-values in *B. cereus* R2F becomes closer to those observed in the class Ib R2F proteins from *M. tuberculosis* [56] and *B. anthracis* [35]. In the higher temperature region, the class Ib R2F-FeIII**_2_**-Tyr^•^ proteins all have lower *P_1/2_*-values relative to class Ia R2's. This trend suggests a weak interaction between the tyrosyl radical and the diiron centre at high temperature. This weaker interaction can be rationalized by longer distance of the tyrosyl radical to the diiron-oxygen cluster in the class Ib proteins, which is observed in their crystal structures [37], [62], [63], [64], [65], [66]. The power saturation behavior of R2F-Mn^III^_2_-Tyr^•^ in presence of NrdI is shown in Figure 7B. The differences in the overall saturation properties are large compared to R2F-Fe^III^_2_-Tyr^•^. The relaxation factor *b* was found to be <1 over the entire temperature range examined and best fit by *b* = 0.6. Attempts to fit the data by forcing constraint on *b* close to unity gave unreliable fits to the microwave power saturation data, thus the recorded trends with *b* = 0.6 indicate the presence of magnetic interaction between the radical and the dimanganese cluster, with a magnetic ground state. Because the overall radical line-width broadening of R2F-Mn^III^_2_-Tyr^•^ compared to R2F-Fe^III^_2_-Tyr^•^, R2F-Mn^III^_2_-Tyr^•^ spectral shape did not change substantially at high temperatures and *b* appears constant and smaller than 1, further supporting a weak dipolar interaction of the radical unit with the ferromagnetic R2F-Mn^III^_2_ cluster. Our EPR signal broadening, as shown previously, was very small, and much smaller than that observed in the R2F-Mn^III^_2_ cluster of *C. ammoniagenes*. The different *P_1/2_* microwave power saturation between the iron and manganese reconstituted RNR R2 proteins are readily explained by weak ferromagnetic coupling of Mn^III^_2_ cluster as observed in *C. ammoniagenes*. In the R2F-Fe^III^_2_-Tyr^•^ system the effective anti-ferromagnetic coupling between the two ferric S = 5/2 centres [62] below 15 K render the magnetic properties of the tyrosyl radical well described as an isolated radical S = 1/2 spin system, since can not be perturbed by nearby effective (with S≠0) spin-systems [62]. On the contrary, the low *b*-factor determined for R2F-Mn^III^_2_-Tyr^•^ extrapolated in the entire temperature range (50–4 K) being accompanied by EPR signal broadening, implies that the tyrosyl radical interacts in *B. cereus* R2F with an additional spin-system (where S≠0) linked to the presence of the ferromagnetic di-manganese core. Consistent with our findings, the earlier work of Cox *et al.* and Cotruvo and Stubbe suggested the presence of a magnetic Mn^III^Mn^III^ cluster at low temperature for the R2F-Mn^III^_2_-Tyr^•^ [20], [26]. It is important to underline that the exact description of the spin multiplicity together with the size of the exchange-energy (*J*) terms among organic/metal spin centres are not yet clear in this system. Our *P*_1/2_–value at 4 K (0.02 mW) clearly shows presence of interaction with a magnetic di-Mn cluster, while at this temperature with the di-Fe protein only show intrinsic tyrosyl-radical behavior without any magnetic interaction to an other center resulting in orders of magnitudes lower *P*_1/2_–value. The *P*_1/2_–values reported for the R2F-Mn^III^_2_-Tyr^•^ from *E. coli* Class Ib are quite different from those observed for *B. cereus* R2F, and have been found much larger in the former [20]. However, the large differences in the extrapolated *b*-factors do not allow direct comparison of these *B. cereus* R2F data with those obtained in R2F proteins from other sources. Our R2F-Mn^III^_2_-Tyr^•^ measurements, on one hand, were performed in presence of NrdI, which increases protein stability, and it is even possible to crystallize a R2F-NrdI complex [23]. On the other hand, the *C. ammoniagenes* and *E. coli* magnetic properties were studied as the R2F-Mn^III^_2_-Tyr^•^without presence of NrdI. Therefore such differences in the samples chemical composition could explain the experimental variation of the R2F-Mn^III^_2_-Tyr^•^ magnetic properties among protein variants, as a result of protein-protein induced changes (e.g. in presence of NrdI) of the tyrosyl-radicals.

### Resonance Raman studies of *B. cereus* R2F reconstituted with Fe

**Figure 8:**
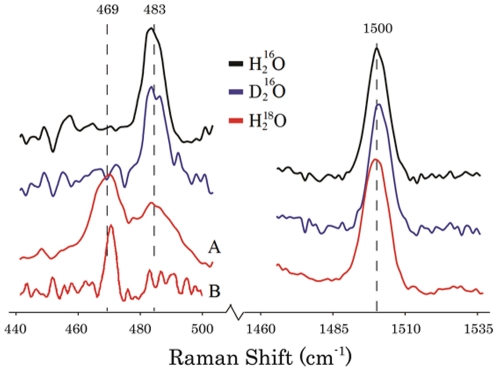
Resonance Raman spectra of the active form of Fe-reconstituted *B. cereus* R2F form. The resonance signature of the Fe-O-Fe vibration region is shown in the left side and the tyrosyl radical C-O vibration is shown in the right side of the panel. Protein recorded in H_2_O (black line) and after isotopic substitutions with D_2_O (blue line) or H_2_^18^O (red line). Methods for isotope substitution are described in the Materials Section. The left side of the panel shows with A) the lower energy region recorded with λ_exc_ = 379.5 nm, 5 mW at the sample and T = 77 K and B) λ_exc_ = 410 nm, 5 mW at the sample and *T* = 77 K. The right side of the panel shows the higher energy region recorded with λ_exc_ = 406.7 nm, 10 mW at the sample and T = 77 K.

Resonance Raman (rRaman) spectroscopy can be employed to unveil minor interactions of the tyrosyl radical with its molecular surrounding. Redox-linked structural changes associated with the electron transfer reaction can be elucidated using vibrational spectroscopy, EPR spectroscopy and by protein crystallography. For instance, tyrosyl radicals e.g., in *E. coli* RNR R2 (Y122^•^) can show movements up to a 1 Å compared to their corresponding not oxidized tyrosine form (Y122) in *E. coli* RNR, *S. typhimurium* or mouse R2 and they have been proven to be coupled to a conformational change in the R2 subunit [65], [67], [68], [69]. The major vibrational fingerprint of the radical unit is the phenoxyl ν_7a_ band (Wilson notation) [70], which contains a major contribution from a C-O^•^ stretching vibration and is a sensitive marker for hydrogen bonding, see our recent study of R2 tyrosyl radical from Epstein-Barr virus [71]. This Raman mode is strongly resonance enhanced by excitation frequencies (λ_ex_) around 405–415 nm, close to the Tyr^•^ absorption maximum (∼410 nm) as shown in Figure 2. The ν_7a_ mode is observed at 1497–1501 cm^−1^ in absence of a hydrogen bond interaction, e.g. *E. coli* R2 [72], while in mouse R2, this value shifts at 1515 cm^−1^ due to hydrogen bonding [40] (Table 2). In mouse R2 a rather close (-O^…^H = 1.89 Å) proton (exchangeable with D_2_O) is present near the tyrosyl oxygen, which was observed with Davies ENDOR experiments [40], [55]. The tyrosyl radical excitation rRaman spectrum of *B. cereus* R2F-Fe^III^_2_-Tyr^•^ (Figure 8, black spectrum) has a peak at 1500 cm^−1^ (λ_exc_ = 407 nm).This Raman mode remains unchanged upon deuterium-oxide exchange (blue spectrum) supporting that no hydrogen bond to the phenoxyl group of the tyrosyl radical is present. Furthermore, theoretical calculation (UB3LYP/6-311++G(d,p)) carried out on the tyrosyl radical under constrained dihedral angle (θ = 60°) well reproduces the experimentally determined energy position of the C-O^•^ vibration (ν_7a_ calc = 1495 cm^−1^) (Calculation S1).

**Table 2:** Resonance Raman parameters for the Fe(III)-O-Fe(III) protein sites and phenoxyl ν_7a_ bands of different RNR proteins.

	ν_s_(Fe-O-Fe) (cm^−1^)			phenoxyl ν_7a_ (cm^−1^)		
Protein	ν	Δ^18^O	Δ^D^	ν	Δ^D^	Ref.
*E. coli* RNR R2 (met and active)	493	−13	+4	1498	0	[72]
Mouse RNR R2	486	−13	−5	1515	−5	[40]
*B. cereus* R2F	483	−14	0	1500	0	This work

The low frequency region of the rRaman spectrum can provide valuable information about the ferric Fe-O-Fe cluster. Proteins with a bent μ-oxo-bridged diferric center have characteristic UV/Vis absorption bands in the 300–450 nm region that are not observed in corresponding μ-hydroxo-bridged proteins or model compounds [7]. The symmetric Fe-O-Fe stretching mode observed around 483 cm^−1^ (Figure 8, black line) is coupled to the oxygen to iron charge transfer transition [7]. This mode displays an ^18^O- induced shift to lower energy in the rRaman spectrum. In the low frequency region (below 1000 cm^−1^) of the active *B. cereus* R2F-Fe^III^_2_ rRaman spectrum, with λ_exc_ at 364 nm or 407 nm, a symmetric vibration at 483 cm^−1^ is observed. This vibrational band is lacking in the rRaman spectra of the apo protein under similar conditions. When the protein was exchanged into H_2_^18^O-water and reconstituted with ^18^O (g), a −14 cm^−1^ shift emerged (Figure 8, red line). The 483 cm^−1^ band could therefore be assigned to the symmetric Fe^III^-O-Fe^III^ stretching mode. Table 2 illustrates a comparison of the Fe-O-Fe vibrations and the phenoxyl ν_7a_ modes for different RNR proteins. The Fe^III^-O-Fe^III^ stretching mode occurs 12 cm^−1^ lower than in *E. coli* R2 and 3 cm^−1^ lower than in mouse R2 suggesting weaker mechanical coupling between the two ferric irons. No change in the Fe-O-Fe vibration was observed in the low energy region of the rRaman spectrum when *B. cereus* R2F was exchanged into D_2_O-containing buffer (Figure 8, blue line). Deuterium oxide incubated mouse R2 has a small 5 cm^−1^ downshift in the frequency associated with the Fe-O-Fe vibration that indicates the presence of a hydrogen bond to the *μ*-oxo bridge [40], thus we have no indication of similar hydrogen bond in *B. cereus* R2F. It has been suggested that the hydrogen bond to the *μ*-oxo bridge could have an influence on the formation of a stable mixed-valence (Fe^III^Fe^II^) cluster. The mixed valence form has been observed by EPR for mouse and herpes simplex virus R2 but not in *E. coli* R2 or *B. cereus* R2F [40], [73], [74]. Presently, we were unable to obtain resolved rRaman spectra of the R2F-Mn^III^_2_-Tyr^•^ form, due to the low radical concentration, stability that the reconstituted protein sample exhibits and presence of the flavin in NrdI with very strong background.

### Conclusion

We have used UV/Vis, X-band, HF-EPR, and rRaman to investigate iron and manganese reconstituted tyrosyl radicals from *B. cereus* R2F. The X-band EPR signal of the tyrosyl radical reconstituted with iron or manganese exhibits some features that are notably different. In R2F-Fe^III^_2_-Tyr^•^, the properties of the tyrosyl radical can be described in terms of a tyrosyl radical not broadened by the iron-cluster below 50 K, only changing the relaxation properties. However, in R2F-Mn^III^_2_-Tyr^•^, the dipolar magnetic interactions between the Tyr^•^ centre and the ferromagnetic manganese- cluster are effective over the whole temperature range examined (from 4 K to 50 K), broadening the tyrosyl radical spectrum and inducing a low *b*-value and high *P_1/2_*. The EPR resonance fingerprint and temperature behavior of R2F-Mn^III^_2_-Tyr^•^ are different from those observed in the Mn forms of *E. coli*, *C. ammoniagenes* and the recently discovered *B. subtilis* [22].This argues for a weaker magnetic interaction between radical and metal sites in *B. cereus*, relative to those in *E. coli* and *C. ammoniagenes* RNR R2 proteins. Differences between the two metal reconstituted forms in *B. cereus* are also reflected in the spread of the *g*-tensor values as well as in the *hfc* with the tyrosyl-radical protons. In addition, we suggest the torsional angle θ slightly increases to 65° in R2F-Mn^III^_2_-Tyr^•^ as compared to R2F-Fe^III^_2_-Tyr^•^ (estimated as 60°). The two forms of reconstituted RNR R2 proteins show differences in chemical stability, with R2F-Fe^III^_2_-Tyr^•^ being most stable. Furthermore, the use of NrdI_ox_ in the Mn reconstitution process was ineffective; only when NrdI_hq_ was present in the sample, the RNR R2 protein could be successfully reconstituted with Mn. HF-EPR and rRaman studies of the R2F-Fe^III^_2_-Tyr^•^ form suggest that the radical is not involved in hydrogen bonding interactions with nearby groups (high g_1_ value and ν_7a_ = 1500 cm^−1^). The specific activities of the Mn-O-Mn core in R2F proteins have been tested recently in both *B. cereus* and *B. anthracis*. The studies evidenced that these systems behaved similarly, showing a raise in protein activity (about eight times) when manganese ions occupy the di-nuclear binding site instead of iron and in presence with the full natural disulfide reductant system [38], [75], [76]. The *B. cereus* R2F-Mn^III^_2_-Tyr^•^ form shows high dependence for the natural R1E reductant NrdH (BC3987 a thioredoxin protein) during activity measurement [38], [75], [76]. These findings reinforce the hypothesis that R2F-Mn^III^_2_-Tyr^•^ form is biologically functional and under some conditions the other Fe-O-Fe form may become active within cell metabolism as well. Our study provides a further example of the spread of finely tuned electronic/magnetic properties associated with both the tyrosyl radical and the catalytic/metal-oxygen binding site present in RNR R2 class Ib proteins.

## Supporting Information

Calculation S1 **Standard thermodynamic quantities (T = 298.15 K and 1.00 atm) calculated by DFT (UB3LYP/6-311++G(d,p), gas phase, EML grid) of the tyrosyl radical, neutral form, doublet.**
(DOC)

Figure S1 **Simulation of the EPR envelope (X-band) of the R2F-Fe^III^_2_-Tyr^•^ from** ***B. cereus*** **R2F by SimFonia software.**
(DOC)

Figure S2 **The HF-EPR (285 GHz) spectrum of R2F-Mn^III^_2_-Tyr^•^ in comparison with the HF-EPR spectrum of R2F-Fe^III^_2_-Tyr^•^, the calculated HF-EPR resolution enhanced R2F-Mn^III^_2_-Tyr^•^ and the HF-EPR of Mn impurity.**
(DOC)
