# Correction: Spectroscopic Studies of the Iron and Manganese Reconstituted Tyrosyl Radical in *Bacillus Cereus* Ribonucleotide Reductase R2 Protein

**DOI:** 10.1371/annotation/0a84fa35-6665-48aa-89e3-88f9d662871b

**Published:** 2012-06-26

**Authors:** Ane B. Tomter, Giorgio Zoppellaro, Caleb B. Bell, Anne-Laure Barra, Niels H. Andersen, Edward I. Solomon, K. Kristoffer Andersson

There was an error in the funding statement. The correct funding is:

This work was supported by The PEOPLE Marie Curie Actions Intra–European Fellowship within the 7th European Community Framework Programme (PIEF–GA–2009–235237 to GZ), the Research Council of Norway (http://www.rcn.no) (Grant 177661/V30, 214239/F20 to KKA and 179513 Leiv Eiriksson travel grant ABT/KKA), the Cancer Foundation of Norway (http://www.kreftforeningen.no) (to KKA), and the NSF-Biochem 0919027 to EIS). The Raman instrument and laser equipment in Oslo was funded by a grant from the Research Council of Norway. The funders had no role in study design, data collection and analysis, decision to publish, or preparation of the manuscript. 

