# Supplementary material for: Spectroscopic Studies of the Iron and Manganese Reconstituted Tyrosyl Radical in Bacillus Cereus Ribonucleotide Reductase R2 Protein
Source: PLoS One. 2012 Mar 14;7(3):e33436. doi: 10.1371/journal.pone.0033436 (PMC3303829; doi:10.1371/journal.pone.0033436)
Supplement: Calculation S1 — Standard thermodynamic quantities (T = 298.15 K and 1.00 atm) calculated by DFT (UB3LYP/6-311++G(d,p), gas phase, EML grid) of the tyrosyl radical, neutral form, doublet. (DOC) [file pone.0033436.s001.doc]

**Supporting Information Calculation S1.**

**Spectroscopic studies of the iron- and manganese reconstituted tyrosyl radical in *Bacillus cereus* ribonucleotide reductase**

**Ane B. Tomter^1^, Giorgio Zoppellaro^1^, Caleb B. Bell III^2^, Anne-Laure Barra^3^, Niels H. Andersen^1^, Edward I. Solomon^2^ and K. Kristoffer Andersson^1^**

^1^Department of Molecular Biosciences, University of Oslo, Oslo, Norway,

^2^Department of Chemistry, Stanford University, Stanford, CA, USA,

^3^Laboratoire National des Champs Magnétiques Intenses, LNCMI-G, UPR 3228, CNRS, Grenoble, France

**Calculation S1.**

**Computational procedure:** geometry optimization (UB3LYP/6-311++G(d,p), in gas-phase), Euler-Maclaurin-Lebedev (EML) grid, containing 70 radial shells with 302 angular points per shell, of the tyrosyl radical (neutral form, doublet, as depicted in the Figure placed on the right) followed by frequency calculation. Below is reported the detailed list of calculated vibrational frequencies and ZPE energies. Due to one constraint on the tyrosine radical backbone (θ = 60°), one imaginary frequency has been found within the list (*). The mode 46 (in red) corresponds to the C-O stretching vibration (ν_7a_ mode, Wilson notation).


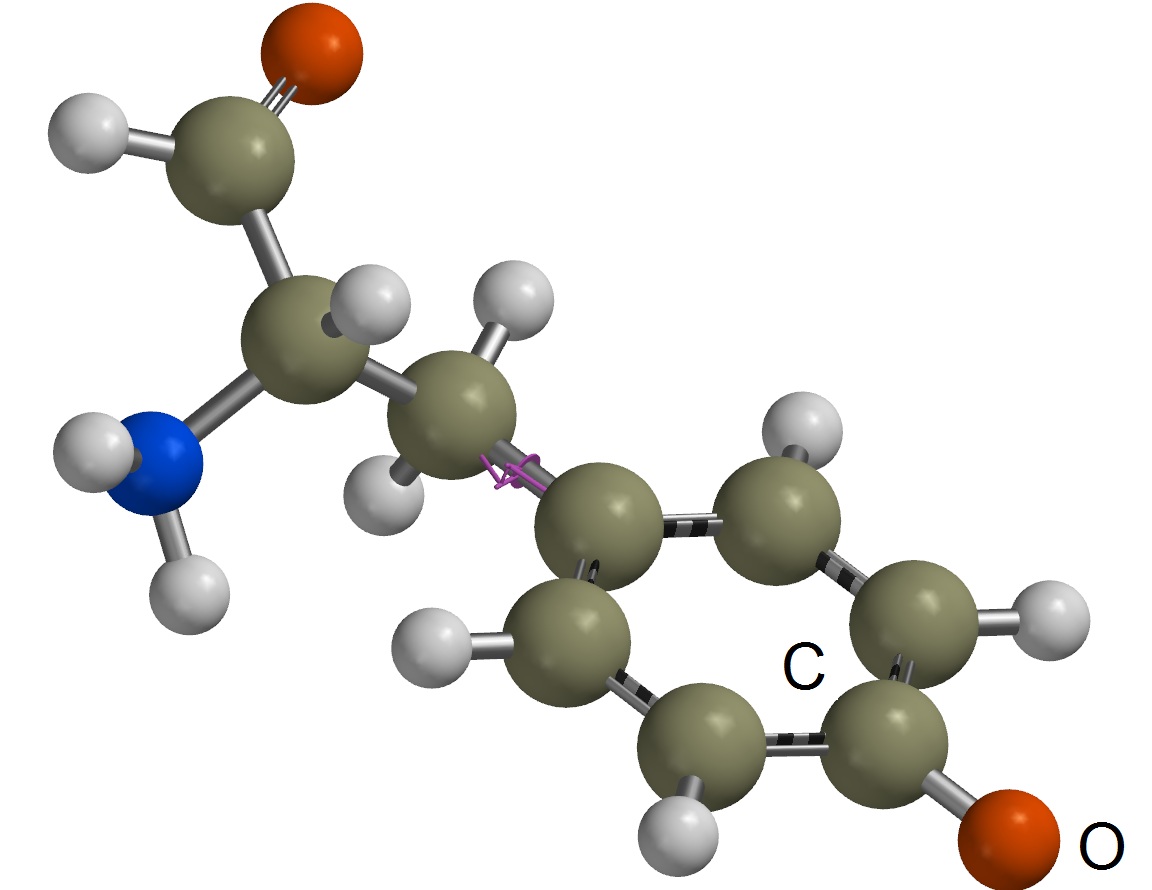


Standard Thermodynamic quantities at 298.15 K and 1.00 atm

Term ZPE Enthalpy Entropy Cv % in

cm-1 kJ/mol kJ/mol J/mol.K J/mol.K Ground IR Int.

-- ---------- ------- ------- ------- ----

1* i 13.096 0.0000 1.2395 8.3144 8.3144 0.00 6.52

2 39.854 0.2384 1.2395 8.3144 8.2888 17.50 10.14

3 68.244 0.4082 1.2395 8.3144 8.2397 28.06 0.94

4 90.332 0.5403 1.2395 8.3144 8.1840 35.33 5.18

5 163.753 0.9795 1.2395 8.3144 7.8949 54.63 15.96

6 184.023 1.1007 1.2395 8.3144 7.7889 58.85 53.78

7 207.062 1.2385 1.2395 8.3144 7.6558 63.18 5.76

8 279.366 1.6710 1.1725 6.4330 7.1618 74.03 2.32

9 321.109 1.9207 1.0355 5.4577 6.8329 78.77 4.16

10 386.209 2.3100 0.8481 4.2457 6.2744 84.49 1.44

11 399.476 2.3894 0.8135 4.0357 6.1556 85.45 5.98

12 437.582 2.6173 0.7208 3.4904 5.8084 87.90 4.55

13 456.769 2.7321 0.6777 3.2449 5.6313 88.97 10.66

14 485.763 2.9055 0.6166 2.9065 5.3622 90.41 11.47

15 544.093 3.2544 0.5080 2.3285 4.8225 92.76 13.46

16 615.727 3.6829 0.3978 1.7714 4.1782 94.88 5.17

17 623.507 3.7294 0.3872 1.7194 4.1102 95.07 0.58

18 714.194 4.2718 0.2811 1.2121 3.3567 96.81 3.67

19 785.870 4.7005 0.2168 0.9167 2.8213 97.75 2.06

20 801.385 4.7933 0.2048 0.8626 2.7131 97.91 0.69

21 812.019 4.8570 0.1969 0.8274 2.6406 98.01 4.93

22 835.241 4.9959 0.1807 0.7551 2.4869 98.22 70.41

23 839.004 5.0184 0.1782 0.7439 2.4626 98.26 52.36

24 871.827 5.2147 0.1576 0.6534 2.2578 98.51 28.35

25 898.216 5.3725 0.1427 0.5884 2.1024 98.69 23.63

26 979.404 5.8581 0.1047 0.4252 1.6749 99.11 19.35

27 980.643 5.8655 0.1042 0.4231 1.6690 99.12 13.76

28 990.562 5.9249 0.1003 0.4066 1.6220 99.16 1.75

29 1006.851 6.0223 0.0942 0.3807 1.5471 99.22 1.60

30 1040.610 6.2242 0.0826 0.3321 1.4008 99.34 6.21

31 1113.148 6.6581 0.0622 0.2472 1.1251 99.54 5.46

32 1125.770 6.7336 0.0591 0.2348 1.0822 99.56 13.93

33 1169.467 6.9950 0.0497 0.1962 0.9442 99.65 0.65

34 1198.113 7.1663 0.0443 0.1744 0.8623 99.69 2.90

35 1219.514 7.2943 0.0407 0.1596 0.8052 99.72 2.25

36 1230.784 7.3617 0.0389 0.1523 0.7765 99.74 1.77

37 1275.956 7.6319 0.0324 0.1263 0.6704 99.79 5.89

38 1294.187 7.7410 0.0301 0.1170 0.6314 99.81 1.39

39 1334.977 7.9849 0.0255 0.0987 0.5514 99.84 12.41

40 1346.159 8.0518 0.0243 0.0942 0.5311 99.85 10.24

41 1392.442 8.3287 0.0201 0.0776 0.4543 99.88 1.54

42 1416.164 8.4705 0.0183 0.0702 0.4189 99.89 14.84

43 1427.527 8.5385 0.0174 0.0669 0.4029 99.90 0.33

44 1440.856 8.6182 0.0165 0.0632 0.3849 99.90 3.61

45 1474.658 8.8204 0.0143 0.0548 0.3424 99.92 5.24

46 1495.025 8.9422 0.0132 0.0503 0.3189 99.93 43.54

47 1506.755 9.0124 0.0125 0.0478 0.3061 99.93 1.25

48 1597.368 9.5544 0.0086 0.0325 0.2220 99.96 105.09

49 1665.405 9.9613 0.0064 0.0243 0.1738 99.97 61.03

50 1814.765 10.8547 0.0034 0.0128 0.1003 99.98 201.10

51 2852.295 17.0605 0.0000 0.0001 0.0017 100.00 144.45

52 2910.513 17.4087 0.0000 0.0001 0.0013 100.00 36.41

53 3019.172 18.0587 0.0000 0.0001 0.0008 100.00 6.95

54 3102.294 18.5558 0.0000 0.0000 0.0006 100.00 3.80

55 3159.025 18.8952 0.0000 0.0000 0.0005 100.00 8.26

56 3176.605 19.0003 0.0000 0.0000 0.0004 100.00 1.72

57 3191.691 19.0905 0.0000 0.0000 0.0004 100.00 5.52

58 3193.572 19.1018 0.0000 0.0000 0.0004 100.00 1.19

59 3499.580 20.9321 0.0000 0.0000 0.0001 100.00 0.20

60 3582.213 21.4264 0.0000 0.0000 0.0001 100.00 4.74

-- ---------- ------- ------- ------- ----

Total Vibrations 455.0874 18.4370 104.4632 152.5699

Ideal Gas 2.4789

Translation 3.7184 172.3547 12.4716

Rotation 3.7184 129.7186 12.4716

---------- ------- ------- -------

Totals 483.4402 406.5365 177.5132

Vibrational(v) Corrections:

Temp. Correction Hv 483.4402

Entropy Correction (Hv-TSv) 362.2314
