# Supplementary material for: Spectroscopic Studies of the Iron and Manganese Reconstituted Tyrosyl Radical in Bacillus Cereus Ribonucleotide Reductase R2 Protein
Source: PLoS One. 2012 Mar 14;7(3):e33436. doi: 10.1371/journal.pone.0033436 (PMC3303829; doi:10.1371/journal.pone.0033436)

**Supporting Information Figure S1**

**Spectroscopic studies of the iron- and manganese reconstituted tyrosyl radical in *Bacillus cereus* ribonucleotide reductase**

**Ane B. Tomter^1^, Giorgio Zoppellaro^1^, Caleb B. Bell III^2^, Anne-Laure Barra^3^, Niels H. Andersen^1^, Edward I. Solomon^2^ and K. Kristoffer Andersson^1^**

^1^Department of Molecular Biosciences, University of Oslo, Oslo, Norway,

^2^Department of Chemistry, Stanford University, Stanford, CA, USA,

^3^Laboratoire National des Champs Magnétiques Intenses, LNCMI-G, UPR 3228, CNRS, Grenoble, France

**Figure S1: Simulation of the EPR envelope (X-band) of the R2F-Fe^III^_2_-Tyr^●^ from *B. cereus* R2F by SimFonia software.** In panel (A) the same *g*-tensor and *A*-tensor (mT units) components with same line width-tensor (LWx,y,z in mT units) as that employed in XSophe has been used. In panel (B) by using larger line-width tensor components as described in Table 1 (main text) satisfactory simulation of the observed EPR envelope was obtained. For convenience, the tables reported in the left of each panel, A and B, show the tensor components (g, A, LW) employed in SimFonia.


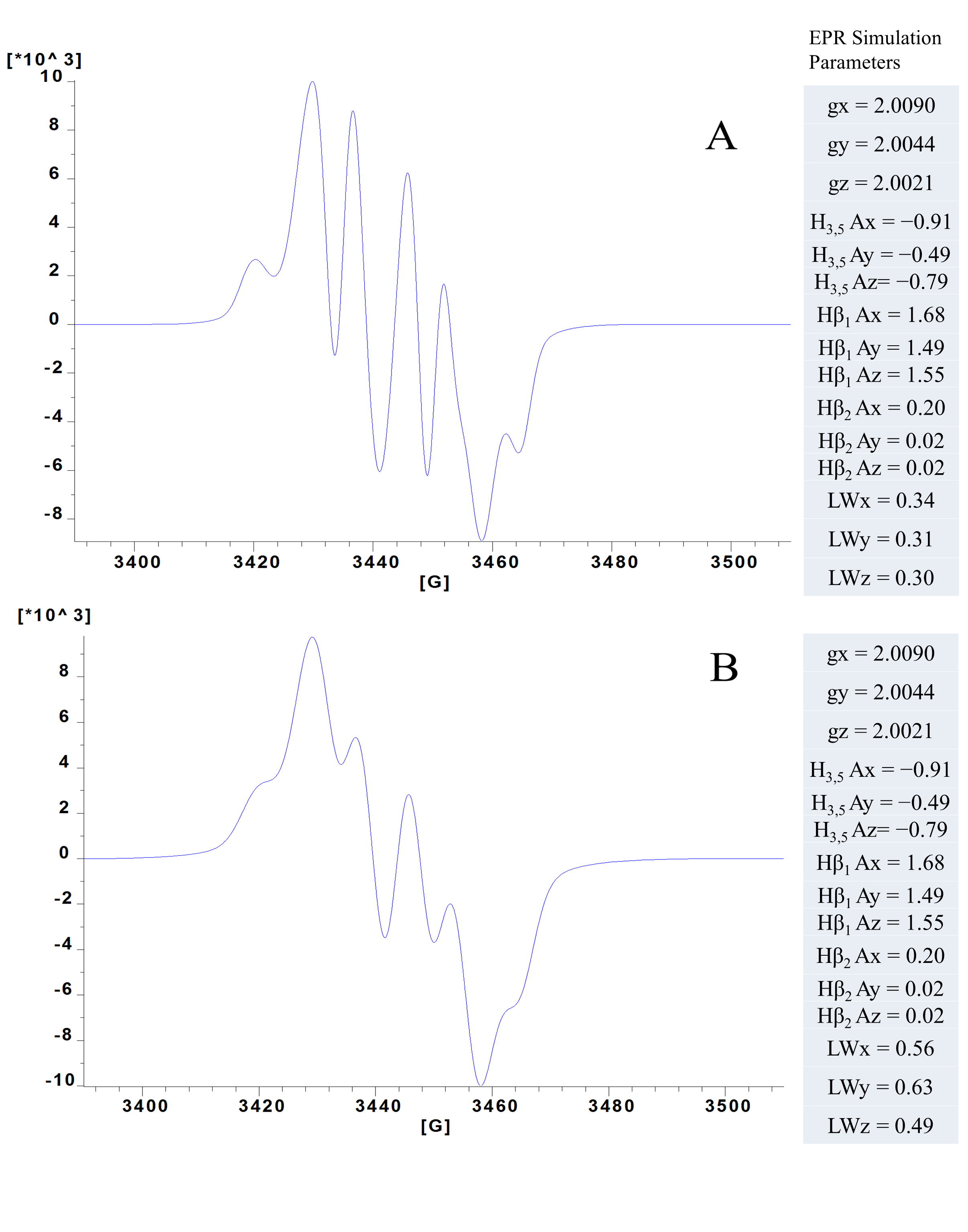

Supplement: Figure S1 — Simulation of the EPR envelope (X-band) of the R2F-FeIII2-Tyr• from B. cereus R2F by SimFonia software. (DOC) [file pone.0033436.s002.doc]
