# Supplementary material for: Spectroscopic Studies of the Iron and Manganese Reconstituted Tyrosyl Radical in Bacillus Cereus Ribonucleotide Reductase R2 Protein
Source: PLoS One. 2012 Mar 14;7(3):e33436. doi: 10.1371/journal.pone.0033436 (PMC3303829; doi:10.1371/journal.pone.0033436)

**Supporting Information Figure S2**

**Spectroscopic studies of the iron- and manganese reconstituted tyrosyl radical in *Bacillus cereus* ribonucleotide reductase**

**Ane B. Tomter^1^, Giorgio Zoppellaro^1^, Caleb B. Bell III^2^, Anne-Laure Barra^3^, Niels H. Andersen^1^, Edward I. Solomon^2^ and K. Kristoffer Andersson^1^**

^1^Department of Molecular Biosciences, University of Oslo, Oslo, Norway,

^2^Department of Chemistry, Stanford University, Stanford, CA, USA,

^3^Laboratoire National des Champs Magnétiques Intenses, LNCMI-G, UPR 3228, CNRS, Grenoble, France

**Figure S2:** The HF-EPR (285 GHz) spectrum of R2F-Mn^III^_2_-Tyr^●^ (top green line) recorded at T = 4.2 K (200 µM R2F) reconstituted in presence of 2X NrdI, with a modulation amplitude of 1.5 mT, 4 accumulations. Due to the low concentration of tyrosyl radical in R2F-Mn^III^_2_-Tyr^●^ (~1/4 of the total protein concentration), the signal-to-noise was lower as compared to R2F-Fe^III^_2_-Tyr^●^. The correspondent HF-EPR spectrum of R2F-Fe^III^_2_-Tyr^●^ obtained under identical conditions is shown for comparison (red line, lowest). The blue second spectrum from top line is a calculated resolution enhanced spectrum of the top green line, using Fourier transform technique described in reference (Twilfer H, Gersonde K, Christahl M (1981) Resolution enhancement of EPR spectra using the Fourier transform technique. Analysis of nitrosyl cytochrome c oxidase in frozen solution. *J Magn Reson*. 44: 470-478) which has been further corrected for Mn(II) impurities. As the Mn(II) impurities overlap the g_2_ and g_3_ resonances of the tyrosyl-radical, these values are not determined with high accuracy. Example of HF-EPR spectrum (285 GHz) of Mn impurity (lower middle ------- orange line) without signal saturation.


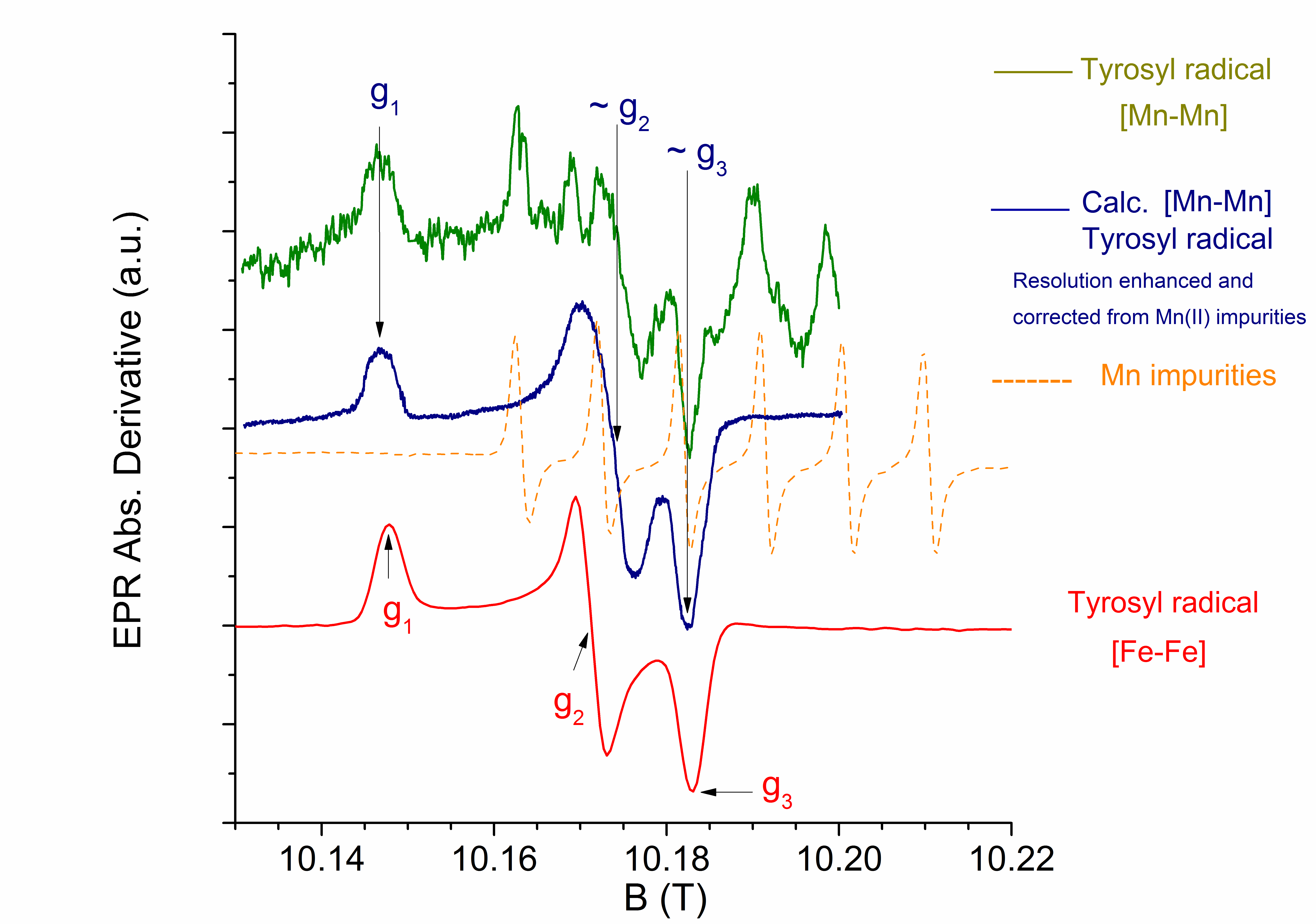

Supplement: Figure S2 — The HF-EPR (285 GHz) spectrum of R2F-MnIII2-Tyr• in comparison with the HF-EPR spectrum of R2F-FeIII2-Tyr•, the calculated HF-EPR resolution enhanced R2F-MnIII2-Tyr• and the HF-EPR of Mn impurity. (DOC) [file pone.0033436.s003.doc]
